# Exploring alternative practice placement models in occupational therapy and physiotherapy: perspectives and experiences of learners and practice educators: a qualitative systematic review

**DOI:** 10.1186/s12909-024-06323-z

**Published:** 2024-11-18

**Authors:** Amanda Deaves, Rebecca Matson, Edwina Rushe, Anna Rees, David Edwards, Kate Trainor, Joanne Seddon

**Affiliations:** 1 School of Allied Health Professions and Nursing, University of Liverpool, Liverpool, England; 2 School of Allied Health, University of Limerick, Limerick, Ireland

**Keywords:** Practice education, Alternative practice placement, Physiotherapy, Occupational therapy, Capacity

## Abstract

Within physiotherapy and occupational therapy practice placements, there is a predominance of the one-to-one supervision model which creates limitations for placement capacity expansion. Alternative placement models must be explored to meet training requirements for these professions and ensure the availability of a future workforce. The aim of this review is to explore the experiences and perceptions of practice educators and learners regarding alternative placement models, to inform future planning and the adoption of these opportunities. This qualitative systematic review followed the Joanna Briggs Institute methodology. A comprehensive search was conducted across six databases (CINAHL, MEDLINE, PubMed, Scopus, ERIC, PsycINFO), covering the period from January 2010 to December 2022. Meta-aggregation was used to synthesise the findings from the included studies. A total of 16 articles were included within this review. The learner voice was represented in 14 categories aggregated to five synthesised findings: professional growth and development, personal and psychological adaptation, alliances within collaborative learning, educational and learning process, and managing professional relationships. The practice educator voice was represented in 11 categories aggregated into four synthesised findings: providing the right support; professional identity; peer relationships and levels of satisfaction. This review highlights positive aspects associated with skill development, personal growth, and the formation of professional identity following alternative placement experiences. However, it also reveals concerns including uncertainty and anxiety related to role clarity, expectations, and the structure within these environments. This understanding of the perceptions and experiences of both learners and practice educators can inform the development of targeted support and guidance from universities, addressing these challenges and enhancing overall satisfaction. Further adoption of alternative placement models has the potential to expand practice placement capacity for learners offering a promising environment for healthcare education. These models provide learners with invaluable experiences and skills essential for their future careers.

**Supplementary Information:**

The online version contains supplementary material available at 10.1186/s12909-024-06323-z.

## Background

An essential requirement of professional registration for occupational therapy (OT) learners globally and physiotherapy (PT) learners within the United Kingdom (UK), is to accrue 1,000 hours of practical experience during training [1, 2]. Practical experiences within practice placements allow learners to develop their skills through workplace practice under the supervision of a clinical educator or mentor [3]. Therefore, achieving the required competencies for practice, across a breadth of lifespan and specialities [1, 2]. Within the UK, obtaining sufficient placement capacity for both professions remains a significant issue due to factors such as balancing contemporary caseload demands, workforce retention, vacant posts and physical space [4, 5]. The allied health workforce must expand to meet future health needs [6–8], therefore increased healthcare learner numbers has led to the exploration of alternative placement models [9, 10]. Occupational therapy and PT are both considered professions under increasing demand but are on the shortage occupation list in a number of countries including the UK, Australia and Canada [11].

The predominance of traditional one-to-one supervision models has created limitations for placement capacity expansion [12]. For this systematic review, alternative placements are defined as any placement model that does not involve one learner per one practice educator. This includes models with multiple students supervised by one practice educator, role emerging placements, leadership and research placements, project focused placements and peer assisted learning. Role emerging placements (REP) explore the profession’s role in a new setting where that profession is not established, with learners supported by an on-site supervisor of a different profession and an off-site profession specific supervisor [13]. Leadership, research and project focused placements involve an identified need related to the placement site that will enable the learner to achieve their learning objectives for the placement such as review of a clinical process or pathway, completion of a clinical audit or production of a service resource [14]. Peer-assisted learning placement focus on collaborative working and learning between the learners whilst supported by one practice educator [15].

While alternative placement models may provide increased autonomy and self-direction, several potential challenges have been identified, particularly regarding their features and reactive implementation [5, 16]. These challenges include increased physical space pressures, lower levels of service user engagement, reduced individual supervision, diminished role identity, and the tendency for these models to be introduced to students at short notice to meet demand, rather than being proactively planned [5, 16].

Previous reviews have included, a mapping review considering practice education models for allied health and social care professionals [5]; a systematic review specific to peer learning and collaborative placement models for healthcare learners [15]; a systematic review considering peer-assisted learning of allied health professionals (AHPs) [16]; a scoping review relating to health profession learners [17]; a systematic review considering technology enhanced learning on clinical placements [18] and a mixed methods literature review considering health professionals as a whole [10]. These reviews have focused on a broader range of allied health and social care professions and have highlighted collaborative peer learning as a key advantage compared to traditional 1:1 placements. However there remains a need for a deeper understanding of the factors that influence the perceived success of more innovative practice education models [18]. The adaptation of alternative placement models necessitates the support, commitment and “buy-in” both learners and practice educators [15, 18]. Therefore, evaluating the factors that contribute to a positive experience is essential.

Concerns regarding learner satisfaction with alternative placement models have been reported [5] highlighting the importance of considering the quality of the experiences whilst expanding placement capacity. Therefore, a focused review of qualitative studies will support increased analysis of educator and learner experiences [17]. Currently no qualitative systematic review has specifically examined OT and PT learner and practice educator experiences and this could provide valuable insights for improving alternative placement model delivery for these healthcare professions.

The professions of OT and PT often work closely within collaborative, patient centred environments and may have overlapping clinical roles [19, 20]. Within the practice environment there can be similarities of OT and PT professional identity and culture as well as communities of practice [21]. The educational pathways for OT and PT students are characterised by an emphasis on functional rehabilitation, interprofessional collaboration, extensive hands-on practice, and a holistic approach to patient care [1, 2]. Learning style preferences can vary across different professional groups within the health sector. However, both OT and PT learners exhibit similar preferences for collaborative and active learning approaches. These preferences are reflected in the academic setting and in the placement environment, where interactive, hands-on learning is prioritised [22, 23]. These elements create a distinct educational experience that prepares graduates for the unique challenges within contemporary practice, differentiating them from other AHPs. Due to these similarities, there is potential for shared educational interactions between the professions [24]. Focusing on these two disciplines will provide insights into how these models are perceived within these professions and whether they require distinct approaches for effective implementation.

The objective of this review is to understand the experiences and perception of learners and practice educators in relation to alternative OT and PT placement models. This will inform the development of future practice placement opportunities thereby helping to address the placement capacity issues, optimise learning and increase sustainability of practice education and workforce development.

### Research question

What are the experiences and perceptions of practice educators and learners, within physiotherapy and occupational therapy, participating in alternative practice placement models?

## Method

The review followed the Joanne Briggs Institute (JBI) qualitative synthesis review approach [25]. This was selected for its established framework in synthesising qualitative research enabling assessment of individual article quality and synthesized evidence strength. Enhancing Transparency in Reporting the Synthesis of Qualitative Research (ENTREQ) checklist was used to guide reporting of this review [26]. See Supplementary File 1.

### Search strategy

A systematic search was undertaken using six electronic databases (CINAHL, MEDLINE, PubMed, Scopus, ERIC, PsycINFO) January 2010 to December 2022. This limited time period sought to ensure that the studies reflected contemporary practice within healthcare education [27]. The search was limited to peer-reviewed studies, published in English, with reference lists of studies also hand-searched. A Boolean search strategy was used to search databases using relevant key terms and alternatives. Key search terms are fully detailed in Table 1.

**Table 1:** Search terms

Population	Phenomena of interest	Context		Limits
Student Learner Mentor Educator “Clinical educator” Physiotherap* “Physical therap*” “Occupational Therap*”	Experience Evaluation View* Attitude* Perception*	“Non-traditional” Blended Collaborative “2 to 1” “3 to 1” “4 to 1” Leadership Coaching “Split placement” “shared placement” “Role emerging” “Project placement” “TECS placement” “Peer learning”	Placement “Practice learning” “Clinical education” “Clinical experience” “Clinical placement”	2010 to current (December 2022) English Language

Rayyan software (www.rayyan.ai) was used to manage references, record decisions, and facilitate audit trail. Titles and abstracts were screened for eligibility by two independent researchers. If this screening failed to define suitability, full-text screening was undertaken. Studies that met inclusion criteria were retrieved and full-text articles were independently reviewed by both researchers. If there was disagreement between the two researchers, a discussion took place with a third researcher to reach agreement.

### Eligibility criteria and study selection

The researchers aimed to include qualitative studies encompassing but not limited to, designs such as phenomenology, grounded theory, ethnography, action research, and interpretive analysis. Mixed-method studies with substantial qualitative components were also considered, specifically those in which the qualitative data were clearly defined and readily extractable from the quantitative data.

The PICo approach (Population, Phenomena of Interest, Context) was used to determine search terms and identify relevant studies.

Population: Studies reporting experiences of OT or PT learners and their practice educators.

Phenomena of interest: studies which explored experiences, views, attitudes or perceptions of learners or practice educators participating in alternative practice placement models.

Context: Alternative practice placement models were defined as including any healthcare setting receiving a pre-registration OT or PT learner implementing skills and behaviours aligned with their respective professions. This includes placements with face-to-face clinical practice experience and remote practice. All settings supporting learner education were included, namely Primary and Secondary care, Health and Social care, Private Practice and Voluntary organisations. Any placement not utilising one-to-one supervision models was also defined as an alternative placement.

### Critical appraisal of studies

Two researchers independently undertook quality evaluation, with disagreements resolved through discussion with a third researcher. The JBI Critical Appraisal Checklist [25] was used to evaluate methodological quality of included studies. Following critical appraisal, all studies scoring less than five on the checklist were excluded in line with the acceptable JBI process [28]. This threshold helps to filter studies of significant methodological flaws or biases which enhances the trustworthiness of the findings [28, 29].

### Data extraction

The JBI Qualitative data extraction tool [25] was utilised within Microsoft Excel. Methods, phenomena of interest, setting, participants, data analysis, cultural information (such as norms, values, or practices that could influence the participants’ experiences), findings and illustrations were retrieved from the studies. Illustrations included examples, quotes, or narrative excerpts from the qualitative data that help to illustrate or support the findings.

Findings from the included studies were extracted using the meta-aggregation approach [26], which is grounded in pragmatism and transcendental phenomenology [31]. Within a meta-aggregative review, the reviewer does not seek to reinterpret the findings of the included studies. Rather, they seek to faithfully present the findings as the original authors intended [25, 30].

Two researchers independently reviewed the studies and identified relevant findings: considering both themes and metaphors. Themes refer to recurring patterns and concepts identified across studies which organise and synthesise data. Metaphors are expressions used by participants to convey symbolic meanings and provide nuanced details into their experiences.

A finding is a verbatim extract of the authors analytic interpretation of their results [25]. The findings were identified by repeatedly re-reading the text. Each finding was accompanied by illustrations that were direct quotes from the participant voice.

Each finding was quantified for credibility as per JBI process (unequivocal, credible and not supported) [31]. Levels of credibility were independently considered by the researchers. The third researcher compared the two independent data extraction results, with disagreements discussed and agreed prior to the finalisation of the data set. All unsupported findings and illustrations were removed from the final data set.

### Data synthesis

Data was synthesised using the three-step process outlined in the JBI manual [31]. To address the research questions, two subgroups were identified: learner voice and practice educator voice. The data synthesis within these subgroups were conducted by two pairs of researchers. Each researcher independently reviewed the data to identify categories of findings, with at least two or more similar findings per category. The meta synthesis involved aggregating these categories into synthesised findings. The two researchers per subgroup (learner voice and educator voice) met to discuss categorises and synthesis, cross checking results, discussing any differences with interpretation and agreeing final categories and synthesised findings. These final meta-aggregation synthesised findings were discussed and agreed by the researcher’s team.

## Results

### Results of the search and quality assessment

**Fig. 1:**
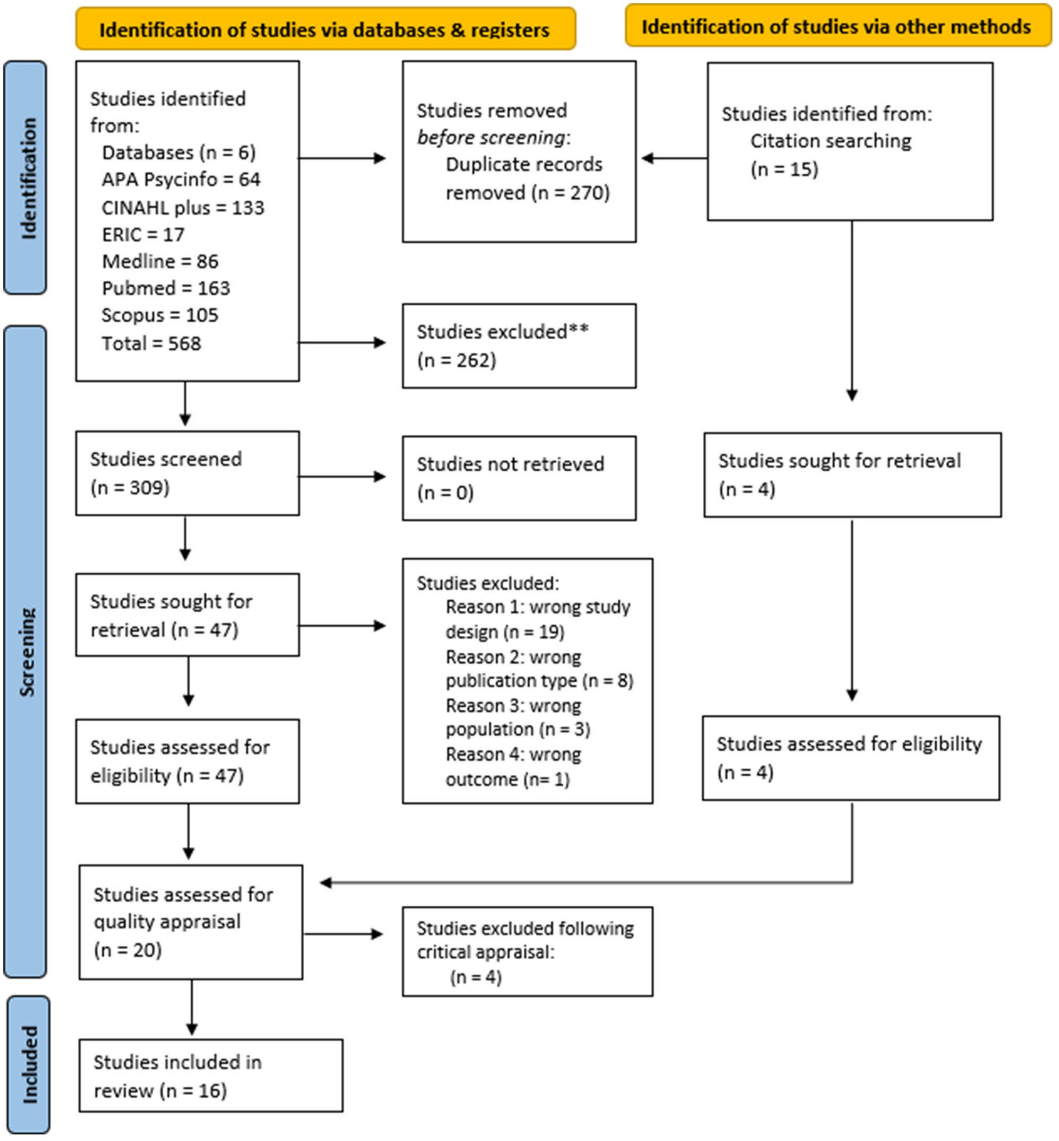
PRISMA 2020 flow diagram for new systematic reviews which included searches of databases, registers and other sources. *From*: Page MJ, McKenzie JE, Bossuyt PM, Boutron I, Hoffmann TC, Mulrow CD, et al.Tetzlaff JM, Akl EA, Brennan SE et al. The PRISMA 2020 statement: an updated guideline for reporting systematic reviews. BMJ 2021; 372:n71. doi: 10.1136/bmj.n71. For more information, visit: http://www.prisma-statement.org/

A total of 568 articles were identified from the six databases and 15 via citation searching. Duplicates were identified (*n* = 270) and removed resulting in 309 records being screened for relevance by title and abstract. In total, 47 full text articles were retrieved for review, of which 20 studies were included. The review and selection process has been recorded in Fig. 1 using the Preferred Reporting Items for Systematic Reviews and Meta-analyses flow diagram [32].

In total, 20 studies met the inclusion criteria and were identified for quality appraisal using the JBI Checklist, shown in Table 2. Following critical appraisal, all studies scoring less than five on the checklist (*n* = 4) were excluded. Quality concerns included a lack of congruity in relation to philosophical perspective, methodology, research question and objectives, data collection methods and data analysis. Therefore, 16 studies were included for data extraction.

**Table 2:** Critical appraisal scoring results

	Authors / Year	Q1	Q2	Q3	Q4	Q5	Q6	Q7	Q8	Q9	Q10	
		Is there congruity between the stated philosophical perspectives and the research methodology	Is there congruity between the research methodology and the research question or objectives?	Is there congruity between the research methodology and the methods used to collect data?	Is there congruity between the research methodology and the representation and analysis of data?	Is there congruity between the research methodology and the interpretation of results?	Is there a statement locating the researcher culturally or theoretically?	Is the influence of the researcher on the research, and vice- versa, addressed?	Are participants, and their voices, adequately represented?	Is the research ethical according to current criteria or, for recent studies, and is there evidence of ethical approval by an appropriate body?	Do the conclusions drawn in the research report flow from the analysis, or interpretation, of the data?	Scores
1	Alpine, Caldas, and Barrett, (2019)	No	No	No	No	No	No	No	Unclear	No	Yes	1.5
2	Barrett, Belton and Alpine, (2021)	No	No	No	No	No	No	No	Yes	Yes	Yes	3
3	Boniface, et al., (2012)	Yes	Yes	Yes	Unclear	Yes	No	No	Yes	Yes	Yes	7.5
4	Clarke, et al., (2014)	Yes	Yes	Yes	Yes	Yes	No	No	Yes	Yes	Yes	8
5	Clarke, et al., ( 2015)	Yes	Yes	Yes	Yes	Yes	Yes	Yes	Yes	Yes	Yes	10
6	Clarke, et al., (2015a)	Yes	Yes	Yes	Yes	Yes	No	No	Yes	Yes	Yes	8
7	Clarke, de Visser and Sadlo, (2019)	Yes	Yes	Yes	Yes	Yes	No	No	Yes	Yes	Yes	8
8	Dancza, et al., (2013)	Yes	Yes	Yes	Unclear	Yes	No	Yes	Yes	Yes	Yes	8.5
9	Dancza, Copley and Moran, (2019)	Yes	Yes	Yes	Yes	Yes	Yes	Yes	Yes	Yes	Yes	10
10	Fortune, and McKinstry, (2012)	Yes	Yes	Unclear	Yes	Yes	No	No	Yes	Yes	Yes	7.5
11	Kaelin and Dancza, (2019)	Yes	Yes	Yes	Yes	Yes	Unclear	Yes	Yes	Yes	Yes	9.5
12	Kyte, Frank and Thomas, (2018)	Yes	Yes	Yes	Yes	Yes	No	Yes	Yes	Yes	Yes	9
13	Mattila and Dolhi, (2016)	No	No	No	No	No	No	No	Yes	No	Yes	2
14	O’Connor, Cahill, and McKay, (2012)	Yes	Yes	Yes	Yes	Yes	Unclear	No	Yes	Yes	Yes	8.5
15	O’Connor, Liston and O’Donnell, (2019)	No	No	No	No	Unclear	No	No	Yes	Yes	Yes	3.5
16	Price and Whiteside, (2016)	Yes	Yes	Yes	Yes	Yes	No	No	Yes	Yes	Yes	8
17	Sevenhuysen, et al., (2015)	No	Yes	Unclear	Yes	Unclear	No	No	Yes	Yes	Yes	6
18	Sharmin, et al., (2016)	Yes	Yes	Yes	Yes	Yes	No	No	Yes	Yes	Yes	8
19	Withers, et al., (2022)	Yes	Yes	Yes	Yes	Yes	Unclear	Unclear	Yes	Yes	Yes	9
20	Wolff-Burke, et al., (2022)	Yes	Yes	Yes	Yes	Yes	No	No	Yes	Yes	Yes	8

## Findings

### Characteristics of the studies

All studies used a qualitative research design; methodological approaches and methods included Interpretative phenomenological analysis (*n* = 4) [33–36]; thematic analysis (*n* = 3) [37–39]; social constructivist thematic analyses (*n* = 3) [40–42]; grounded theory (*n* = 2) [43, 44]; interpretive description (*n* = 2) [45, 46]and action research (*n* = 2) [47, 48].

Eight studies explored the views of the learners alone: OT (*n* = 7) [33–36, 40, 43, 47] and PT (*n* = 1) [38]. Seven studies explored the views of both the learners and the practice educators; OT (*n* = 3) [41, 45, 48], PT (*n* = 3) [39, 44, 46]and both OT and PT (*n* = 1) [37]. One study reported the practice educator voice alone [42].

Geographical location of the studies included Ireland (*n* = 2) [37, 40], UK (*n* = 7) [33–36, 38, 47, 48], Australia (*n* = 3) [39, 42, 43], Canada (*n* = 2) [45, 46], Switzerland (*n* = 1) [41] and USA (*n* = 1) [44].

The range of alternative placement include REPs (*n* = 11) [33–36, 38, 40, 41, 45–48], project placements (*n* = 1) [43], 2:1, peer assisted learning and collaborative learning (*n* = 4) [37, 39, 42, 44]. The study characteristics can be found in Table 3.

Four of the included papers are from one study completed by the same research authorship group [33–36], whilst all other studies report unrelated qualitative research activities.

**Table 3:** Characteristics of included studies

Authors / Year	Country / Culture	Aims or Purpose of the Study	Study Methodology / Method	Phenomenon of interest	Setting	Participants	Data analysis
Boniface, et al., (2012)	Setting is UK, but students are from UK and Poland / European	Explore experiences of staff and students using pyramidal model of peer-assisted and long-arm supervisors in role-emerging practice placement	Participatory Action Research / interviews	Student and supervisors’ experiences of peer-assisted learning and long arm supervision	Role emerging placements	Two final year postgraduate diploma occupational therapy students from a UK university, two occupational therapy students from a Polish university, UK university staff (OTs)	Reflexive TA (Braun and Clarke)
Clarke, et al., (2014)	Brighton- UK / UK	Gain deeper understanding of occupational therapy students’ experience of role-emerging placements	Phenomenological approach / interpretative phenomenological analysis / interviews	Student experience of role emerging placements	Role emerging placements	Five female MSc pre-registration occupational therapy students	IPA
Clarke, et al., (2015)	UK (Brighton) / UK	Explore the impact of role-emerging placements on professional practice and identity once graduated	Phenomenological approach / Interpretative phenomenological analysis (IPA) / interviews	Professional practice and identity	Role emerging placements	Five female MSc pre-registration occupational therapy graduates	IPA
Clarke, et al., (2015a)	Brighton – UK / UK	Explore occupational therapy students’ experience of role-emerging placements	Phenomenological approach / Interpretative phenomenological analysis / interviews	How occupational therapy students experienced and ascribed meaning to their role-emerging placements.	Role emerging placements	Five OT students (pre-reg Masters)	IPA
Clarke, de Visser and Sadlo, (2019)	Brighton – UK / UK	To explore strategies used by students to cope on role-emerging placements	Phenomenological / interpretative phenomenological analysis / semi-structured interviews	How occupational therapy students cope with the challenges of undertaking a role-emerging placement.	Role emerging placements	Five female students (pre-reg MSc in OT)	IPA
Dancza, et al., (2013)	Ireland and England / European	To explore students’ perspectives of their learning in a variety of role-emerging placement settings	Social constructionism / Interviews	Learning experiences	Role emerging placements	10 OT students	Reflexive TA (Braun and Clarke)
Dancza, Copley and Moran, ( 2019)	UK / UK	Exploring the learning experiences of students (from both supervisor and student perspectives) during role-emerging placement.	Action research methodology / reflective field notes, placement documentation and semi-structured interviews	Exploration of the learning experiences of students, from both supervisor and student perspectives, over the duration of a role-emerging placement in schools, to contribute to our understanding of this important student learning process.	Role emerging placements	14 students (OT), 5 on-site supervisors, 3 long arm supervisors, 1 researcher/ long arm supervisor	Template analysis
Fortune, and McKinstry, (2012)	La Trobe University, Australia / Australia	Exploring the perceptions of agency sponsors and students participating in a project placement.	Grounded theory approach / open-ended, written questionnaires & interviews	Value of project placements	Project Placement - project occurs in place where OT services do not exist.	11 project sponsors, 11 final year OT masters students	Adapted grounded theory approach with themes identified
Kaelin and Dancza, (2019)	Zurich – Switzerland / Switzerland	Examine how occupational therapy students on role-emerging placements in school settings experience applying threshold concepts and how impacts onto placement	Social constructivist paradigm / semi-structured focus group interviews	Experience of applying threshold concepts	Role emerging placements	13 female Swiss occupational therapy students, one long-arm supervisor for all student	Template analysis
Kyte, Frank and Thomas, (2018)	UK (research conducted in University of Worcester) / UK	Explore the experiences of physiotherapy students who had undertaken a role-emerging placement	Qualitative / semi-structured focus groups	Experiences of undergraduate physiotherapy students who had each undertaken a REP	Role emerging placements	Six physiotherapy students	Thematic analysis
O’Connor, Cahill, and McKay, (2012)	Ireland / Ireland	Compare experiences and perspective of clinical educators and students involved in 1:1 and 2:1 models of placement supervision and the learning opportunities available with both models	Qualitative - descriptive approach / individual semi-structures interviews	Perspectives of clinical educators and students (OT and PT) who had participated in both the 1:1 (one student supervised by one clinical educator) and 2:1 (two students supervised by one clinical educator) models of clinical education	Clinical education (PT and OT) − 1:1 and 2:1 supervision model	24 students (OT and PT) and 29 educators	Thematic content analysis
Price, and Whiteside, (2016)	Melbourne (Australia) / Australia	Determine the experience of supervisors pre-and-post 2:1 supervision model experience	Social constructivist theoretical perspective / focus group	Supervisor experience in the trial of a new 2:1 supervision model (OT)	Health service OT department	Nine occupational therapy supervisors	Thematic Analysis
Sevenhuysen, et al., (2015)	Australia / Australia	To explore clinical placement experiences of students and clinical educators in paired peer-assisted learning model, compared to traditional paired teaching approach	Qualitative study / focus groups	Experiences of students and clinical educators in a paired student placement model incorporating facilitated peer-assisted learning (PAL) activities, compared to a traditional paired teaching approach	Traditional paired model and a PAL paired model.	24 physiotherapy students and 12 clinical educators.	Thematic Analysis
Sharmin, et al., (2016)	Southern Ontario, Canada / Canada	To explore the benefits and challenges of engaging student occupational therapists in HIV service organisations through role-emerging placements.	Interpretive description / interviews	Benefits and challenges of role-emerging placements of student occupational therapists in HIV service organisations	Roleemerging placements	Five student occupational therapists (5), (2) staff members from the HIV service organisations (3) and an off-site occupational therapy preceptor (1) - total of 11	Content analysis
Withers, et al., ( 2022)	Ontario (Canada) / Canada	To understand the experiences of students, clinical instructors, health care providers and patient in role-emerging placement and identify barriers and facilitators.	Interpretive description y / semi-structured interviews	Experiences and perspectives of a role emerging placement in an emergency department	Roleemerging placements	51 participants (6 PT students, 1 clinical instructor, 15 nurses, 12 physicians, and 17 patients)	Thematic analysis in an interpretive description
Wolff-Burke, et al., (2022)	USA - West Virginia / USA	Examine the perspectives on processes that occur in students and clinical educators throughout CCLE and to expand on factors that influence a successful CCLE	Grounded Theory / surveys and focus groups / journals and focus groups	Clinical collaborative learning experience (CCLE) - factors perceived in influencing the success of this, examination of the processes that make this successful	Clinical collaborative learning experience (multiple students paired with one clinical instructor)	29 DPT students and 18 Clinical Instructors	Grounded Theory approach / Thematic analysis

## Meta-synthesis of the findings

### Meta-synthesis of learner voice

A total of 142 findings, classed as unequivocal or credible, were extracted from 15 articles about learner experience and aggregated into 14 categories based on the similarity of meanings.

Five synthesised findings were developed from these categories: professional growth and development, personal and psychological adaptation, alliances within collaborative learning, educational and learning process, and managing professional relationships (Table 4).

**Table 4:** Contribution of studies to meta-synthesis of learner voice

Synthesised findings	Professional growth and development			Personal and psychological adaptation				Alliances within collaborative learning		Educational and learning process			Managing professional relationships	
Category	Developing professional identity	Development of professional role and skills	Person centered care	future employability	Mixed emotions	Anxiety and isolation	Enhance personal values & confidence	Challenges with peer assisted learning	Value of peer assisted learning	Learning experience and environments	Learning process and theoretical frameworks	Supervision process	Building relationships and teamwork	Managing expectations
Boniface et al., (2012)	*	*						*	*	*		*	*	*
Clarke et al., (2014)	*		*				*							
Clarke et al., (2015)	*		*	*			*							
Clarke et al., (2015a)	*	*				*				*				*
Clarke, de Visser and Sadlo (2019)	*	*					*		*		*		*	
Dancza et al., (2013)	*	*			*	*	*		*	*	*	*	*	*
Dancza, Copley and Moran, (2019)						*			*	*	*	*		
Fortune and McKinstry, (2012)		*			*					*		*	*	
Kaelin and Dancza, (2019)			*				*			*	*			*
Kyte, Frank and Thomas, (2018)		*	*	*	*	*	*		*	*		*		*
O’Connor, Cahill, and McKay, (2012)							*	*		*	*	*		
Sevenhuysen et al., (2015)					*				*		*	*	*	
Sharmin et al., (2016)		*	*						*				*	
Withers, et al., (2022)							*			*	*	*	*	
Wolff-Burke, et al., (2022)									*			*	*	

#### Professional growth and development

Professional growth and development centres around the learner building essential knowledge, skills, identity, values, and behaviours to become a competent professional. This synthesised finding evolve around the learners development journey and highlights the impact of the placement environment in facilitating this professional growth.

##### Developing professional identity

Alternative placement models enabled learners to better understand professional behaviours and values by articulating their role and identity as an OT [33–36, 40, 47].

"I came in with a much clearer idea of my role; the placement definitely helped me to clarify that."

(Sophie, p46) [34].

##### Development of professional roles and skills

The placement models have allowed the learners to enhance their understand of their professional roles and the remit of their professional boundaries. The REP allowed learners to increase their understanding of their professional role and utilise these skills within unique environments [38, 45, 48].

“Other healthcare professionals that are working there try to push you out of your remit of what you’re meant to be doing […] I think maybe establishing that [your role] before and asking your placement educator “.

(Participant 3, student, p4) [38].

Learners valued opportunities to become proficient in profession-specific skills and developed confidence [34, 36, 38, 40, 43, 45, 47].

*“The role-emerging placement did give me the confidence to work. Because you are so independent in a role-emerging placement*,* and it really forces you to use your critical thinking skills and draw upon the knowledge that you have learned over two years in the occupational therapy programme.”*

(Student OT 5, p 576) [45].

##### Person-centred care

The individual was central to the learner’s decision-making and actions [33, 34, 38, 41, 45]. The learners demonstrated understanding of this core value by ensuring the individual was the focal point of their professional behaviours.

"For me, [a highlight] was also the client-centeredness. We really assessed what the teacher needed and what was important to her. Then, we really set goals together and worked towards them. At the end we even were successful!"

(Andrea, p715) [41].

##### Future employability

Learners expressed mixed views regarding the impact of alternative models on future employability, reflecting diverse experiences and outlooks regarding employment readiness [34, 38].

"Working with the homeless in the future would be great; I’d love to do something like that, looking at different roles.health promotion would be really cool as well, I think. Occupational therapists could work really well in health promotion."

(Jayne, p47) [34].

"We don’t know how employers are going to view physios doing role-emerging placements, and you don’t know what they’re going to think of that when they see that."

(Participant 1, student, p7) [38].

#### Personal and psychological adaptation

This finding highlighted emotional challenges faced during placements, including heightened anxiety and isolation. Personal development was also emphasised and encompassed self-growth, values and enhance confidence in their abilities.

##### Mixed emotions

Challenges learners encountered during alternative placements led to both positive and negative emotions [34, 38, 40, 43].

"It has been such a roller coaster between loving and hating the placement!"

(Michelle, during the placement, p432) [40], .

The feeling of being overwhelmed was familiar, and tiredness was induced by enhanced and diverse cognitive demands [34, 38, 40, 43].

"It just felt, felt very overwhelming and yeah there were a couple of days that week when I thought ‘this is too much, what have I done?"

(Sophie, p37) [34].

##### Anxiety and isolation

Feelings of anxiety and stress compared to peers on traditional placements were an area of concern [34, 38, 40, 48].

*“I was concerned with this placement*,* that obviously*,* being third year that*,* all my friends would have different*,* more specific placements*,* and my knowledge would kind of stay static. And I wouldn’t really learn that much from it*."

(student, p3) [38].

The isolation of working alone within placement areas without broader team support was expressed explicitly about REPs [34, 38].

"I really am out there on my own, that would be the only thing I would say, I felt a little bit out there on my own and thrown in, you know."

(student, p34) [34].

##### Enhanced personal growth and confidence

These findings reflect the learner’s development related to their own values, personal characteristics and enhanced confidence. Despite challenges within alternative placements, learners recognised and reflected upon individual personal growth [33, 35, 36, 40].

".... it was kind of like a learning curve for myself as well’ cause my personal development, not just professional but me as a person [thinking] ‘yeah, I am grown up enough to do that sort of thing; I’m not little anymore."

(Jayne, p224) [35], .

Enhanced confidence among learners was expressed throughout multiple aspects of skills and practice-based categories [33, 34, 36–38, 41, 46].

. I grew in my confidence of what I was doing, what actually OT is, how you could sort of implement interventions, how you can assess people. I grew practically like that but also .just with my self-belief as well about my own abilities.

(Jayne p225) [33].

#### Alliances within collaborative learning

This explores the challenges as well as the value of peer-assisted learning. These experiences revolve around the dynamics of the relationships and activities in a peer learning environment.

##### Challenges with peer-assisted learning

Challenges included difficulties with navigating relationship dynamics, issues with independent marking, complexities in providing feedback to other learners and differences in learning styles [37, 39, 47, 48].

"I felt we were being marked very much as a pair…."

(ST3PT; 2:1, p279) [37].

"We got used to giving each other feedback, and now we still do that even though we don’t have to . So, I guess sometimes you might think you don’t want to tell them offend them, but because we had to in the beginning now, we just keep giving each other feedback."

(student, FG2) [39], .

##### Value of peer-assisted learning

Learners within REP pairs valued each other’s support, with the collaborative models enabling peer-support that enhanced practice and reduced anxiety [36, 38–40, 44, 45, 47, 48].

*“I learnt quite a lot from her*,* I would ask her why she did things in different ways*,* in her approach she was quite daring and wanted to give things a try and I think in the past I would be the kind of person to hold back … “*.

(Cardiff student, p199) [47].

[40]*“Peer supervision was great as well*,* being able to share our journey and how we were feeling. And if one of us was feeling low then the other one was able to pick you up. I think*,* to be honest*,* you actually do need two students.”*

(Student 4, p572) [48].

#### Educational and learning process

This synthesised finding captured the learners’ experiences of learning and supervision during placements, reflecting upon the learning activities, process and environment that impacted their experience. Learners recognised the challenges of the discomfort of learning and the theory-practice connections.

##### Learning experience and environments

Learning progression was considered slow, with limited opportunities within the placement [37, 41].

[41]*“The only negative was that the caseload was so small because it was divided between us… you probably didn’t get as much contact with patients because of the 2:1 model…that was the one thing I suppose… was the setting suitable for a 2:1 model?”*

(ST10OT; 2:1, p280) [37].

However positive feelings related to ‘belonging with the placement’ and the ability to ‘think for themselves’ was valued. While REP raised concerns regarding unfamiliar environments and client groups, these settings were also seen as valuable [35, 38, 40, 41, 43, 46–48].

"I really value the independent learning and being responsible for my own learning [.] whenever I’ve done that, I’ve shown my best qualities."

(Participant 6, p5) [38].

#####  Learning process and theoretical frameworks

OT learners within REPs indicated these placements provided solid opportunities for utilising theoretical frameworks [41, 48].

"We learned [about occupation-centred practice] theoretically, and yeah, we knew what it was, but this time, we really used it in practice and saw how the different steps [in the occupational therapy process] can be done and that they actually make sense and that they are needed. Yeah, it gave me this understanding, the practical side actually."

(Elin, p714) [41].

Learners valued learning tools and reflective activities embedded within the placement structure [36, 37, 39, 41, 46].

*“It’s great*,* because you get a lot more time for self-evaluation and self-reflection. You’re on your own a lot*,* but you get used to that*,* and that’s real life*,* right? . [The clinical instructor] is great*,* too. He’s always available by phone [for questions]. It’s mostly self-directed*,* but there is still supervision that you can turn to if you need it.”*

(Student 03 p283) [46].

##### Supervision process

The learners’ express preferences related to the type and style of supervision they preferred from both their placement educators and university staff. Within REPs, closer and more frequent supervision was preferred [38–40, 43, 44, 46–48].

*“I think it’s important that Uni supervisors accept me if they are not too busy – mine was not available as much as I wanted. I only met with the supervisor at the start and end.…but it was a waste of time because she was too busy.*

*(student p268)* [43].

#### Managing professional relationships

Interprofessional and interpersonal relationships are a core component within the practice learning environments. Learners developed skills relating to maintaining and developing these relationships and valuing teamwork.

##### Building relationships and teamwork

The learners recognised that relationships required careful negotiation, and a sense of belonging and effective teamwork was valued alongside client relationships [36, 40, 44, 45].

"I was just part of the team, and I said to them at the end, ‘I’m really grateful ’cause you didn’t make me feel like a little student who didn’t have a clue; you just let me crack on; I was part of the team, I had a valued contribution."

(Ella, p23) [36].

However, managing perceived hierarchy was challenging [43, 46].

"Working with team members with fixed attitudes, support of these members as long as they were asked not to participate. Delegating tasks, this was particularly difficult as student and an outsider to the org."

(Student, p269) [43].

##### Managing expectations

The REP provoked concerns regarding expectations from clients, services, and the learners themselves. Some learners found the lack of clarity a challenge [47] and managing the expectations of the staff was particularly demanding [34, 38, 40].

"What was mentally draining was trying to manage everyone’s expectations as well as doing something for myself and my own learning…."

(Ursla, p431) [40].

### Meta-synthesis of the practice educator voice

A total of 49 findings were extracted from nine articles exploring the practice educator’s voice. These findings were aggregated into 11 categories based on similarity of meaning. Four synthesised findings were developed from these categories: providing the right support, professional identity, peer relationships, and levels of satisfaction (Table 5).

**Table 5:** Contribution of studies to meta synthesis of practice educator’s voice

Synthesised findings	Providing the right support			Professional Identity		Peer relationships			Levels of satisfaction	
**Category**	**Support requirements**	**Supervisor feedback**	**Additional support structures**	**Clinical reasoning development**	**Role clarity and expectations**	**Partnering for peer growth**	**Independence**	**Collaborative working**	**Satisfaction with the model**	**Dissatisfaction with the model**
Boniface et al., (2012)	*	*			*					
Dancza, Copley and Moran, (2019)				*						*
Kaelin and Dancza, (2019)	*		*	*	*		*			
O’Connor, Cahill, and McKay, (2012)						*				*
Price and Whiteside, (2016)	*		*			*	*	*	*	*
Sevenhuysen et al., (2015)	*	*				*		*	*	*
Sharmin et al., (2016)					*					*
Withers, et al., (2022)									*	
Wolff-Burke et al., (2022)	*							*		*

#### Providing the right support

This synthesised finding relates to the different approaches that alternative placement models may require to help ensure success reflecting adjustments and additional elements to a more traditional placement for both the learner and the educator.

##### Support requirements

Identifying the level of support means tailoring different styles and levels of support depending on learners’ needs, which can include greater pastoral support [39, 41, 42, 44, 47].

"Some students were really good, took a lot of initiative and we didn’t have to ask a lot of questions at all. We had others that needed more prompting."

(educator, FG4) [39].

"I think you have to create that safe space for both of those students so they share those fears and concerns."

(CI4 FG P2, p149) [44].

Alternative placement models allowed supervisors to develop different skills and approaches, which benefited their professional development, however practice educators desired support for themselves [47].

"at least one of the LAS [long-arm supervisors] needs to be experienced because I was also being supervised to a certain extent by the other LAS."

(Supervisor, p199) [47].

##### Supervisor feedback

The variety and significance of supervisor feedback for the learner has been recognised by practice educators particularly the high value in relation to the assessment aspects [39, 47].

"[Students] want to know they’re doing well from their [educator] because they’re the ones that are going to assess them."

(educator, FG5) [39].

##### Additional support structures

Successful placements were related to the university’s role in matching learners with placement settings, along with additional information related to learners needs and skill level [41, 42].

"It was good to be able to meet with the teachers before the students started their placement. It allowed to talk about what students experienced the last year and to talk about expectations and student learning."

(long-arm supervisor p715) [41].

#### Professional identity

Practice educators noted challenges and opportunities in relation to the growth of professional identity for the learners, in relation to the structure, process and role within the setting.

##### Clinical reasoning development

Clinical reasoning was enhanced within the alternative models, meeting the learner’s expectations for practice, and allowing a more client-centred approach [41, 48].

"On previous placements, the students were just told what to do and they just did it without thinking about it."

(Long arm supervisor, p570) [48].

"it seemed like their approach on the role-emerging placement was closer to really client-centred occupational therapy practice…."

(long arm supervisor, p716) [41].

The utilisation of a specific framework: the Occupational Therapy Intervention Process Model (OTIPM) [49, 50] was perceived as beneficial to guide the learner’s approach and facilitate the develop of their reasoning to support the intervention within one study [48].

"By using the OTIPM to structure the approach of students it puts the brakes on…."

(Long arm supervisor, p572) [48].

##### Role clarity and expectations

Practice educators reflected that learners need guidance on the remit of their roles, especially within REPs, and recognised their own uncertainty regarding learner independence prior to professional registration [41, 45, 47].

"we need to be really much clearer about what we expect each person to do … about what level of supervision each person has."

(Supervisor, p199) [47].

"It was unclear at times how far you could proceed with the client, given the limitation that they are not occupational therapists; they are just students."

(OT preceptor, p578) [45].

#### Peer relationships

Having more than one learner within a placement was both beneficial and challenging in terms of professional relationships and development opportunities.

##### Partnering for peer growth

Peer working resulted in greater leaner independence, productivity, and confidence. This supported skill development in delivery of feedback which is essential for practice, leadership and reducing demands on practice educators [37, 39, 42].

"If there are two students, they can bounce things off each other, and there is a lot of shared learning, so I always felt that it is better with 2 students."

(CE6PT; 2:1, p279) [37].

*“If we’re skilling our students to give feedback to each other*,* I think it’s a good skill to have when they are coming to clinical practice."*

(Educator FG4, p89) [39].

##### Independence

Learners could become co-dependent and “didn’t want to be separated” [42] (p125), and challenges were identified within placements without on-site supervision, but positive reflection was noted [41].

"They reflected how this might be similar to working as an occupational therapist after graduation and how it was a valuable learning experience."

(Long arm supervisor, p715) [41].

##### Collaborative working

The placement model led to development of teamwork and professionalism, as learners were required to work together and adjust to the needs of others [39, 42, 44].

"The more experienced student was helping to lead and guide the more novice student. They learned to rely on each other, which was a great dynamic."

(CL10 FG P1, p149) [44].

Some practice educators queried the rationale and evidence behind the peer-working approach [42].

"I want to know if there is actually research that is showing that it is an effective way of learning for students."

(supervisor, p 127) [42].

#### Levels of satisfaction

This related to the differing demands and benefits that alternative placement models provide.

##### Satisfaction with the model

The perceived benefits, influencing practice educators to consider alternative placements include reduced need for practice educator feedback and learner productivity, which decrease practice educator workload, along with unique opportunities that the alternative placement models can provide [39, 42, 46].

"These students [administered] . 150 to 175 new assessments each. There was endless opportunity for learning with regards to patient history taking, documentation, collaboration, and communication with the interprofessional team… so many aspects of the placement that offered learning opportunities that do not exist in other placement settings."

(Clinical instructor, p282) [46].

##### Dissatisfaction with the model

Additional resource demands such as physical space, experiences and administration (including paperwork, preparation and time) could make practice educators reluctant to offer alternative placements [37, 39, 42, 44, 45, 48].

*“I think from a preparation point of view*,* it’s a little bit more difficult to try to*.

*arrange the diary to facilitate two students”*.

(CE5PT;2:1, p280) [37].

## Discussion

Placement capacity is a recognised concern [5], yet the potential benefits of alternative practice education models to address or enhance this demand have not been thoroughly explored [10]. This systematic review investigates the experiences and perceptions of practice educators and learners regarding alternative placement models. The synthesised findings reveal shared analytical themes that could promote the adoption of these models, increasing training capacity and supporting workforce expansion [9]. There are four main areas of focus identified within this review; development of professional identity, skill development, psychological safety and support, and the potential impact of alternative models.

Professional identity is shaped through defining professional boundaries, multidisciplinary teamwork, and facilitation of safe and effective clinical practice for health professionals [51]. The key influencers in this development are the individuals themselves, and the impact of relationships with clients and other professionals [52]. The synthesised findings of ‘Professional growth and development’ and ‘Professional Identity’ found that REPs, in particular, supported learners to develop strong professional identity and perceived confidence in their abilities, a finding consistent within the research [53]. Both learners and practice educators highly valued opportunities for professional growth. Occupational therapy has long recognised and established this growth within REPs [55]. Our review highlights the benefits of this model for physiotherapy learners, and it suggests that this approach could be extended to other healthcare professions [18, 56].

The professional skill development opportunities were valued by learners and the practice educators. Learners were more focused on specific practical skill, whereas practice educators indicated that clinical reasoning, as a higher-level skill, could be enhanced within alternative placement models. Learning environments that promote independent learning processes around uncertainties and active critical analysis support the development of clinical reasoning processes [54]. Our review suggests that structured, well-organised learning environments with experiential learning opportunities were highly valued. Autonomous placement models, such as REPs or 2:1 models, can enhance clinical reasoning professional socialisation and learning processes [55, 56]; therefore boosting learner confidence. In contrast, more traditional placements often dictate choices based on organisational or practice educator preferences [57]. Whilst previous literature has predominately focused on nursing and medicine, it is acknowledged that all placement models offer valuable opportunities for skill development among AHP learners [5, 17]. Our review supports this perspective for OT and PT professions, indicating potential opportunity in relation to the workforce and placement challenges among this group. Alternative placement models have the capacity to provide the OT and PT learners with the culture and environment to facilitate the integration of professional knowledge and skills, aligned with findings of existing literature [5, 58].

The synthesised findings of ‘Personal and psychological adaption’ and ‘Providing the right support’ has highlighted the significance of psychological safety in ensuring the learner’s well-being and the requirement of careful consideration when planning and supporting the learners within these environments. Psychological safety encompasses both intrapersonal and interpersonal factors [59] Therefore, consideration of emotional intelligence and resilience to support educational safety allows learners to engage without risk [60]. Our review suggests that focusing upon how to support psychological safety, within the placement structure may enhance the successful adoption of these models. Enablers of psychological safety within healthcare teams have included organisational, team and individual factors [59]. Therefore, HEI’s could implement activities that enhance learner understanding and preparation prior to and during these alternative placements, reducing the psychological impact of uncertainty as reported in previous literature [58, 61]. Working in partnership with practice educators could address the areas of practice where the educators feel less confident and would value more support [12, 62]. Confidence in the support provided, along with the need to adequately prepare all parties, is essential [11, 15]. Our review highlights the need to enhance practice educator training provided by the universities, facilitate an educational culture within the placement settings and adequately prepare learners regarding expectations, which could reduce anxieties for all stakeholders.

A range of potential positive impacts of alternative placement models have been identified within our review; these include employability, peer learning and inclusive learning approaches. Within healthcare literature employability is often discussed in terms of skills and capabilities [63]. However, a flexible and changeable mindset could better manage unpredictability and uncertainty within the changing landscapes of healthcare [64]. It could be suggested that these models, due to the personal growth opportunities, better prepare graduates for the challenges of the future employment market. Wider literature reports that REPs enhance skills, improve employability and support career development [53] with graduates experiencing more positive outcomes in skills, career pathways and employment status [13]. The practice placement experiences for nursing and healthcare students can have influence upon future employment choices [65, 66] therefore nurturing these positive relationships and connections could be beneficial for all stakeholders.

Peer learning and collaboration were valued by the learners and the practice educators within our review, consistent with previous findings that these models enhance confidence and teamwork skills within healthcare learners [15]. However, the nature of these peer relationships can be complex, leading to challenges within the placement environment [67]. Challenges identified included a range of cultural aspects such as unconscious biases, patient contact time, and physical space, all consistent within recent literature [16, 68]. Effective strategies are required to address the barriers and facilitators to maximise the peer learning opportunities within these alternative placement models.

Our review has highlighted that inclusive learning approaches are influenced by the practice educator and the learner/educator relationship. Learners highly regard practice educators who build relationships, promote active participation, and are engaged in communities of practice, therefore supporting the learner’s professional development [62]. These themes of belonging, acceptance, supervision and support are not unique OT and PT and to these alternative placements but are valued within all placement models for healthcare learners [66]. A successful placement experience is characterised by the ability to connect with practice educators and clients, thereby building valuable relationships within the learner/educator dynamic [15, 69]. These connections entail establishing trust, fostering active participation, and emphasising outcome-based learning [70]. The sense of belonging, community of learning practice and role models are effective methods of supporting learning and increase satisfaction [5, 62, 71]. Inclusive learning approaches can be developed within alternative placements therefore facilitating a successful experience for all.

Our review has highlighted that to support the adoption of these alternative placements the benefits, challenges and expectations of these placements needs to be clarified for all stakeholder’s pre-placement by the university. There appears to be ambiguities and uncertainties that are contributing to challenges for both practice educators and learners. Addressing these issues and emphasising the potential for developing higher-level clinical reasoning skills as well as enhancing employability, may foster improved psychological well-being and better integration within placements. By understanding the perceptions and experiences of OTs and PTs, universities can provide targeted profession-specific support and guidance that addresses these concerns with the aim to increase the culture and acceptance of alternative placement models.

### Strengths and limitations

Utilisation of the JBI meta-aggregation process promotes a rigorous approach to the method. The adoption of this approach allowed for the combining of findings that is more than the sum of the individual findings aligned with that of meta-analysis [25]. The critical appraisal, standardised data extraction and synthesis approached allowed for a clear and robust processes to draw recommendations and conclusion related to the research question.

Limitations within this qualitative review are related to challenges of identifying all relevant studies, due to the date range and grey literature search restrictions. The search was completed December 2022 and studies after this date have not been included. Grey literature was not included to maintain rigor as this may have introduced challenges in assessing methodological quality. There was the potential for qualitative viewpoints from OT’s or PT’s reported within multi-professional studies to have been missed due to the limitations of the search strategy. The search terms focused specifically on OT and PT, while ensured a targeted relevant literature, it may have resulted in the exclusion of qualitative data from multi-professional studies that involved OT and PT alongside other health professions. Only studies in English were included. All the research included were conducted pre the global COVID pandemic which may well have had an impact on the current experiences of learners and practice educators.

Whilst 16 articles were included within the data for analysis, four data sets were from the same participants from a body of work from one authorship group. Eleven studies considered REP, 10 explored OT placements in comparison to four exploring PT placements: limiting the breadth of data available in terms of scope of participants and range of alternative practice placement models. This highlighted the lack of literature related to the scope of alternative placements within OT and PT and limits the generalisability of these findings beyond REP.

The JBI process was followed to ensure rigour in the extraction and aggregation of the findings however, qualitative meta-synthesis involves individual interpretation and decision-making during knowledge production. A team approach was utilised to ensure consensus was achieved, via repeated reading of the articles and discussion to ensure that the synthesised findings were grounded in the data. All reviewers were lecturers who were either Occupational Therapists or Physiotherapists, each with interest and experience in practice education and placement provision. Reviewers worked collaboratively in the interpretation and reporting of the qualitative data and considered their prior assumptions, experiences and professional backgrounds during team discussions.

### Future research

Future studies are required to explore the views within different alternative models to build on the understanding related to these experiences particularly within the physiotherapy profession in relation to REP’s. Consideration of the employability factors related to the range of skills developed within the alternative placement models would enhance understanding related to the potential benefits. Focusing upon strategies and techniques to enhance these opportunities for learners, practice educators and organisations has the potential to facilitate increases in placement opportunity and capacity. As this study has focused on OT and PT further consideration to explore these alternative models in the broader range of AHPs and nursing could provide other profession specific perspectives to support the capacity challenges within healthcare education.

## Conclusion

This review synthesised the experiences of OT and PT learners and practice educators within alternative placement opportunities. Alternative placement models can present a positive environment to support the development of professional identity and personal growth, providing clear benefits in terms of clinical reasoning, professional skills and person-centred care. While these models present additional emotional challenges, prioritising psychological safety can foster confidence and support personal growth among learners. Peer learning and collaboration further enhance these opportunities, contributing to skill development. Additional support and guidance from the university and managing learner expectations were emphasised when utilising such models. The success of these models hinges on targeted support, preparation and ongoing guidance for both learners and practice educators. Recommendations include strengthening these support process to maximise the benefits, prevent uncertainty and support the psychological safety of the learner and practice educator. These findings emphasise the multifaceted nature of alternative placements in healthcare education and highlight the importance of addressing challenges to maximise the learner experience. Nonetheless, these models have the potential to enable increased practice placement capacity for learners. Overall, alternative placements offer a promising environment for healthcare education, proving learners with invaluable experiences and skills essential for their future careers.

## Electronic supplementary material

Below is the link to the electronic supplementary material.

Supplementary Material 1

## Data Availability

Data is provided within the manuscript or supplementary information files.

## Abbreviations

AHPs: Allied Health Professions
ENTREQ: Enhancing Transparency in Reporting the synthesis of Qualitative research
JBI: Joanne Briggs Institute
OT: Occupational Therapy
OTIPM: Occupational Therapy Intervention Process Model
PT: Physiotherapy
PICo: Population, Phenomenon of Interest, Context
REP: Role Emerging Placement
UK: United Kingdom

## References

[1] Minimum Standards for the Education of Occupational Therapists Revised. [https://wfot.org/resources/new-minimum-standards-for-the-education-of-occupational-therapists-2016-e-copy]

[2] Accreditation of qualifying programmes in. physiotherapy quality assurance processes. [https://www.csp.org.uk/documents/csp-accreditation-qualifying-programmes-physiotherapyqualityassuranceprocesses]

[3] Standards of education and training guidance. [www.hcpc-uk.org/globalassets/meetings-attachments3/plg---standards-of-education--training-review/2016/march/enc-03---standards-of-education-and-training-2014-version/].

[4] Craik C, Turner A. A chronic shortage of Practice Placements: whose responsibility? Br J Occup Therapy. 2005;68(5):195–6.

[5] Beveridge J, Pentland D. A mapping review of models of practice education in allied health and social care professions. Br J Occup Therapy. 2020;83(8):488–513.

[6] Alsafi Z, Abbas A-R, Hassan A, Ali MA. The coronavirus (COVID-19) pandemic: adaptations in medical education. Int J Surg. 2020;78:64–5. 32304892 10.1016/j.ijsu.2020.03.083 PMC7156945

[7] Practice placements. challenges and solutions [https://www.hee.nhs.uk/our-work/allied-health-professions/increase-capacity/practice-placements-challenges-solutions]]

[8] Salter C, Oates RK, Swanson C, Bourke L. Working remotely: innovative allied health placements in response to COVID-19. Int J Work-Integrated Learn. 2020;21(5):587–600.

[9] Hellawell M, Graham C, O’Brien C. Is practice placement capacity helping the NHS to recruit healthcare professionals? Br J Healthc Manage. 2018;24(4):198–202.

[10] Millington P, Hellawell M, Graham C, Edwards L. Healthcare practice placements: back to the drawing board? Br J Healthc Manage. 2019;25(3):145–53.

[11] Helping to ensure an essential supply of allied health professions. Practice placements challenges and solutions. [https://www.hee.nhs.uk/sites/default/files/documents/Ensuring%20an%20essential%20supply%20-%20Oct2020.pdf].

[12] Barrett EM, Belton A, Alpine LM. Supervision models in physiotherapy practice education: student and practice educator evaluations. Physiother Theory Pract. 2021;37(11):1185–98. 31782324 10.1080/09593985.2019.1692393

[13] Syed S, Duncan A. Role Emerging Placements: skills Development, Postgraduate Employment, and Career pathways. Open J Occup Therapy. 2019;7(1):12.

[14] Overton A, Clark M, Thomas Y. A review of non-traditional occupational therapy practice placement education: a focus on role-emerging and project placements. Br J Occup Therapy. 2009;72(7):294–301.

[15] Markowski M, Bower H, Essex R, Yearley C. Peer learning and collaborative placement models in health care: a systematic review and qualitative synthesis of the literature. J Clin Nurs. 2021;30(11–12):1519–41. 33461240 10.1111/jocn.15661

[16] Sevenhuysen S, Thorpe J, Molloy E, Keating J, Haines T. Peer-Assisted Learning in Education of Allied Health Professional Students in the clinical setting: a systematic review. J Allied Health. 2017;46(1):26–35. 28255594

[17] Nyoni CN, Dyk LH-V, Botma Y. Clinical placement models for undergraduate health professions students: a scoping review. BMC Med Educ. 2021;21(1):1–598. 34863178 10.1186/s12909-021-03023-w PMC8642754

[18] Jones A, Wilson I, McClean S, Kerr D, Breen C. Supporting the learning experience of health-related profession students during clinical placements with technology: a systematic review. Rev Educ. 2022;10(2):e3364.

[19] De Wit L, Putman K, Lincoln N, Baert I, Berman P, Beyens H, Bogaerts K, Brinkmann N, Connell L, Dejaeger E. Stroke rehabilitation in Europe: what do physiotherapists and occupational therapists actually do? Stroke. 2006;37(6):1483–9. 16645135 10.1161/01.STR.0000221709.23293.c2

[20] Trojanowski S, Woodworth J, Wiencek AR, Yorke A. An introduction to interprofessional education for first semester doctoral occupational therapy and physical therapy students. Internet J Allied Health Sci Pract. 2021;19(2):9.

[21] Snell R, Fyfe S, Fyfe G, Blackwood D, Itsiopoulos C. Development of professional identity and professional socialisation in allied health students: a scoping review. Focus Health Prof Education: Multi-Professional J. 2020;21(1):29–56.

[22] Olivier B, Jacobs L, Naidoo V, Pautz N, Smith R, Barnard-Ashton P, Ajidahun AT, Myezwa H. Learning styles in physiotherapy and occupational therapy students: an exploratory study. South Afr J Occup Therapy. 2021;51(2):39–48.

[23] Brown T, Cosgriff T, French G. Learning style preferences of occupational therapy, physiotherapy and speech pathology students: a comparative study. Internet J Allied Health Sci Pract. 2008;6(3):7.

[24] Cleary KK, Howell DM. The educational interaction between physical therapy and occupational therapy students. J Allied Health. 2003;32(2):71–7. 12801018

[25] Chapter. 2: Systematic reviews of qualitative evidence.

[26] Tong A, Flemming K, McInnes E, Oliver S, Craig J. Enhancing transparency in reporting the synthesis of qualitative research: ENTREQ. BMC Med Res Methodol. 2012;12(1):181–181. 23185978 10.1186/1471-2288-12-181 PMC3552766

[27] Helbach J, Pieper D, Mathes T, Rombey T, Zeeb H, Allers K, Hoffmann F. Restrictions and their reporting in systematic reviews of effectiveness: an observational study. BMC Med Res Methodol. 2022;22(1):230. 35987985 10.1186/s12874-022-01710-w PMC9392276

[28] Butler A, Hall H, Copnell B. A guide to writing a qualitative systematic review protocol to Enhance evidence-based practice in nursing and Health Care. Worldviews Evidence-based Nurs. 2016;13(3):241–9. 10.1111/wvn.12134 26790142

[29] Shaheen N, Shaheen A, Ramadan A, Hefnawy MT, Ramadan A, Ibrahim IA, Hassanein ME, Ashour ME, Flouty O. Appraising systematic reviews: a comprehensive guide to ensuring validity and reliability. Front Res Metr Anal. 2023;8:1268045. 38179256 10.3389/frma.2023.1268045 PMC10764628

[30] Hannes K, Lockwood C. Pragmatism as the philosophical foundation for the Joanna Briggs meta-aggregative approach to qualitative evidence synthesis. J Adv Nurs. 2011;67(7):1632–42. 21466579 10.1111/j.1365-2648.2011.05636.x

[31] Aromataris ELC, Porritt K, Pilla B, Jordan Z, editors. JBI Manual for Evidence Synthesis. *Available from*: https://www.synthesismanualjbiglobal 2024.

[32] Page MJ, McKenzie JE, Bossuyt PM, Boutron I, Hoffmann TC, Mulrow CD, Shamseer L, Tetzlaff JM, Akl EA, Brennan SE, et al. The PRISMA 2020 statement: an updated guideline for reporting systematic reviews. Syst Reviews. 2021;10(1):89–89. 10.1186/s13643-021-01626-4 PMC8008539 33781348

[33] Clarke C, Martin M, Sadlo G, de-Visser R. The development of an authentic Professional Identity on Role-Emerging Placements. Br J Occup Therapy. 2014;77(5):222–9.

[34] Clarke C, Martin M, de Visser R, Sadlo G. Sustaining professional identity in practice following role-emerging placements: opportunities and challenges for occupational therapists. Br J Occup Therapy. 2015;78(1):42–50.

[35] Clarke C, Martin M, Sadlo G, de-Visser R. ‘Facing uncharted Waters’: challenges experienced by occupational therapy students undertaking role-emerging Placements. Int J Practice-based Learn Health Social Care 2015:30–45.

[36] Clarke C, De-Visser R, Sadlo G. From trepidation to Transformation: strategies used by Occupational Therapy Students on Role-Emerging Placements. Int J Practice-based Learn Health Social care. 2019;7(1):18–31.

[37] O’Connor A, Cahill M, McKay EA. Revisiting 1:1 and 2:1 clinical placement models: student and clinical educator perspectives. Aust Occup Ther J. 2012;59(4):276–83. 22934900 10.1111/j.1440-1630.2012.01025.x

[38] Kyte R, Frank H, Thomas Y. Physiotherapy Students’ experiences of Role-Emerging Placements: a qualitative study. Int J Practice-based Learn Health Social Care. 2018;6(2):1–13.

[39] Sevenhuysen S, Farlie MK, Keating JL, Haines TP, Molloy E. Physiotherapy students and clinical educators perceive several ways in which incorporating peer-assisted learning could improve clinical placements: a qualitative study. J Physiotherapy. 2015;61(2):87–92. 10.1016/j.jphys.2015.02.015 25801365

[40] Dancza K, Warren A, Copley J, Rodger S, Moran M, McKay E, Taylor A. Learning experiences on role-emerging placements: an exploration from the students’ perspective. Aust Occup Ther J. 2013;60(6):427–35. 24299482 10.1111/1440-1630.12079

[41] Kaelin VC, Dancza K. Perceptions of occupational therapy threshold concepts by students in role-emerging placements in schools: a qualitative investigation. Aust Occup Ther J. 2019;66(6):711–9. 31514234 10.1111/1440-1630.12610

[42] Price D, Whiteside M. Implementing the 2:1 student placement model in occupational therapy: strategies for practice. Aust Occup Ther J. 2016;63(2):123–9. 26831283 10.1111/1440-1630.12257

[43] Fortune T, McKinstry C. Project-based fieldwork: perspectives of graduate entry students and project sponsors. Aust Occup Ther J. 2012;59(4):265–75. 22934899 10.1111/j.1440-1630.2012.01026.x

[44] Wolff-Burke M, Fergus A, Ferrone D, Moulder A, Thompson K, Whitley J. The collaborative clinical learning experience in physical therapy: student and instructor perspectives. J Phys Therapy Educ. 2022;36(2):146–53.

[45] Sharmin R, Jung B, Shimmell L, Solomon P. Benefits and challenges of role-emerging placements of student occupational therapists in HIV service organisations. Int J Therapy Rehabilitation. 2016;23(12):574–82.

[46] Withers J, Zavitz C, Nguyen T, Baglole J, Kashetsky N, Graham E, Brison R, Law M, Booth R, Miller J. Experiences of Physiotherapy Students, Health Care providers, and patients with a role-emerging Student Clinical Placement in an Emergency Department: a qualitative study. Physiotherapy Can. 2022;74(3):278–86. 10.3138/ptc-2020-0040 PMC10262821 37325219

[47] Boniface G, Seymour A, Polglase T, Lawrie C, Clarke M. Exploring the nature of peer and Academic Supervision on a role-emerging Placement. Br J Occup Therapy. 2012;75(4):196–201.

[48] Dancza K, Copley J, Moran M. Occupational therapy student learning on role-emerging placements in schools. Br J Occup Therapy. 2019;82(9):567–77.

[49] Fisher AG, Marterella A. Powerful practice: a model for authentic occupational therapy. Fort Collins, Colorado: Center for Innovative OT Solutions; 2019.

[50] Fisher GA. Occupational therapy intervention process model: a model for planning and implementing top-down, client-centered, and occupation-based interventions. Fort Collins, Colorado: Three Star; 2009.

[51] Matthews J, Bialocerkowski A, Molineux M. Professional identity measures for student health professionals - a systematic review of psychometric properties. BMC Med Educ. 2019;19(1):308–10. 31409410 10.1186/s12909-019-1660-5 PMC6693256

[52] Cornett M, Palermo C, Ash S. Professional identity research in the health professions—a scoping review. Adv Health Sci Education: Theory Pract. 2023;28(2):589–642. 10.1007/s10459-022-10171-1 PMC10169899 36350489

[53] Thew M, Thomas Y, Briggs M. The impact of a Role Emerging Placement while a student occupational therapist, on subsequent qualified employability, practice and career path. Aust Occup Ther J. 2018;65(3):198–207. 29527692 10.1111/1440-1630.12463

[54] Furze J, Black L, Hoffman J, Barr JB, Cochran TM, Jensen GM. Exploration of students’ clinical reasoning development in Professional Physical Therapy Education. J Phys Therapy Educ. 2015;29(3):22–33.

[55] Gruppetta M, Mallia M. Clinical reasoning: exploring its characteristics and enhancing its learning. Br J Hosp Med. 2020;81(10):1–9. 10.12968/hmed.2020.0227 33135931

[56] Moores AV, Dancza KM, Turpin MJ, Copley JA. The nature of theory used in Practice Education: a scoping review. Can J Occup Therapy (1939). 2022;89(3):261–82. 10.1177/00084174221093466 35635132

[57] Ashby S, Chandler B. An exploratory study of the Occupation-focused models included in Occupational Therapy Professional Education Programmes. Br J Occup Therapy. 2010;73(12):616–24.

[58] O’Brien A, McNeil K, Dawson A. The student experience of clinical supervision across health disciplines – perspectives and remedies to enhance clinical placement. Nurse Educ Pract. 2019;34:48–55. 30458410 10.1016/j.nepr.2018.11.006

[59] O’Donovan R, McAuliffe E. A systematic review of factors that enable psychological safety in healthcare teams. Int J Qual Health Care. 2020;32(4):240–50. 32232323 10.1093/intqhc/mzaa025

[60] Cleary M, Visentin D, West S, Lopez V, Kornhaber R. Promoting emotional intelligence and resilience in undergraduate nursing students: an integrative review. Nurse Educ Today. 2018;68:112–20. 29902740 10.1016/j.nedt.2018.05.018

[61] Cant R, Ryan C, Hughes L, Luders E, Cooper S. What helps, what hinders? Undergraduate nursing students’ perceptions of Clinical Placements based on a thematic synthesis of literature. SAGE Open Nurs. 2021;7:23779608211035845. 34782862 10.1177/23779608211035845 PMC8590386

[62] O’Connor DA, Baird T, Jack K, Wilkinson RG, Chambers A, Hamshire C. Supporting physiotherapy learners in practice settings: a mixed methods evaluation of experiences of physiotherapy educators. Physiother Theory Pract. 2023;40(8):1–14. 37259912 10.1080/09593985.2023.2219313

[63] Leadbeatter D, Nanayakkara S, Zhou X, Gao J. Employability in health professional education: a scoping review. BMC Med Educ. 2023;23(1):33–4. 36650469 10.1186/s12909-022-03913-7 PMC9844949

[64] Bates G, Rixon A, Carbone A, Pilgrim C. Beyond employability skills: developing professional purpose. J Teach Learn Graduate Employab. 2019;10(1):7–26.

[65] Wareing M, Taylor R, Wilson A, Sharples A. Impact of clinical placements on graduates’ choice of first staff-nurse post. Br J Nurs. 2018;27(20):1180–5. 30418848 10.12968/bjon.2018.27.20.1180

[66] Rowland E, Trueman H. Improving healthcare student experience of clinical placements. BMJ Open Qual 2024, 13(1). 10.1136/bmjoq-2023-002504 PMC10773407 38176708

[67] Tailor J, Wadsworth H, McCallig M, Horobin H. Student physiotherapists’ perspectives of peer learning during multi-model placements. Physiotherapy. 2024;123:102–8. 38447496 10.1016/j.physio.2024.02.004

[68] Tai JHM, Canny BJ, Haines TP, Molloy EK. Implementing peer learning in Clinical Education: a Framework to address challenges in the Real World. Teach Learn Med. 2017;29(2):162–72. 27997224 10.1080/10401334.2016.1247000

[69] Cassidy E, Norris M, Williams A. What does it take to graduate? A qualitative exploration of the perceptions of successful physiotherapy graduates from one university in the UK. Physiother Theory Pract. 2020;36(2):316–32. 29913096 10.1080/09593985.2018.1485799

[70] Sellberg M, Halvarsson A, Nygren-Bonnier M, Palmgren PJ, Möller R. Relationships matter: a qualitative study of physiotherapy students’ experiences of their first clinical placement. Phys Therapy Reviews. 2022;27(6):477–85.

[71] Jack K, Hamshire C, Chambers A. The influence of role models in undergraduate nurse education. J Clin Nurs. 2017;26(23–24):4707–15. 28334475 10.1111/jocn.13822

